# Epidemiology and drug resistance analysis of bloodstream infections in an intensive care unit from a children’s medical center in Eastern China for six consecutive years

**DOI:** 10.1007/s10123-024-00481-2

**Published:** 2024-01-18

**Authors:** Huijiang Shao, Xin Zhang, Yang Li, Yuanyuan Gao, Yunzhong Wang, Xuejun Shao, Ling Dai

**Affiliations:** 1 Department of Clinical Laboratory, Children’s Hospital of Soochow University, No. 92, Zhong Nan Street, Industrial Park, Suzhou, 215025 China; 2 Institute of Pediatric Research, Children’s Hospital of Soochow University, Suzhou, 215025 China

**Keywords:** Children, Pathogen, ICU, Bloodstream infection, Drug resistance, MDR bacteria

## Abstract

**Background:**

Children in the intensive care unit (ICU) who suffer from severe basic diseases and low immunity are usually in critical condition. It is crucial to assist clinicians in selecting the appropriate empirical antibiotic therapies for clinical infection control.

**Methods:**

We retrospectively analyzed data from 281 children with bloodstream infection (BSI). Comparisons of basic data, pathogenic information, and drug resistance of the main bacteria were conducted.

**Results:**

We detected 328 strains, including Gram-positive bacteria (223, 68%), mainly coagulase-negative *Staphylococci* (CoNS); Gram-negative bacteria (91, 27.7%); and fungi (14, 4.3%). The results of the binary logistic regression analysis showed that the main basic disease was an independent risk factor for death. Compared with *Escherichia coli*, *Klebsiella pneumoniae* exhibited a higher proportion of extended-spectrum β-lactamases (ESBLs), and its resistance to some β-lactamides and quinolones antibiotics were lower. Twenty-seven isolates of multidrug-resistant (MDR) bacteria were detected, of which carbapenem-resistant *Acinetobacter baumannii* (CRAB) accounted for the highest proportion (13, 48.2%).

**Conclusions:**

CoNS was the principal pathogen causing BSI in children in the ICU of children, and *Escherichia coli* was the most common Gram-negative pathogen. The main basic disease was an independent risk factor for death. It is necessary to continuously monitor patients with positive blood cultures, pay special attention to detected MDR bacteria, and strengthen the management of antibiotics and prevention and control of nosocomial infections.

**Supplementary Information:**

The online version contains supplementary material available at 10.1007/s10123-024-00481-2.

## Introduction

Bloodstream infection (BSI) is a systemic infectious disease caused by pathogenic microorganisms entering the blood system and can manifest as bacteremia or even sepsis (Gouel-Cheron et al. 2022). Sepsis is a serious systemic inflammatory reaction and is one of the main causes of death in children (Zhang et al. 2022). Children in the intensive care unit (ICU), often in critical condition with severe basic diseases and low immunity, are prone to infections (Yan et al. 2021). According to statistics, the hospital infection of children in the ICU is approximately 2–5 times that of children in general wards (Bassetti et al. 2015; Bammigatti et al. 2017). Once a child suffers from BSI, it not only aggravates the illness and elevates pain but also leads to extended hospital stays, significantly inflating medical expenses. This situation seriously threatens the life of the child and amplifies the financial burden on the family (Tran et al. 2017; Zhu et al. 2019). Recently, the incidence and mortality rates of BSI have remained high. Studies have shown that BSI is the most common hospital-acquired infection in the ICU, with a mortality of 18.6–52.3% (Shime et al. 2012; Marsillio et al. 2015; Schwab et al. 2018; Markwart et al. 2020). Therefore, early and appropriate antibiotic therapy can improve the prognosis of children with sepsis admitted to the ICU. Blood culture is considered the most effective laboratory method for diagnosing BSI. In clinical practice, pathogens are identified through blood culture results, and rational drug use is based on drug sensitivity results. However, the low positive rate of blood cultures and delayed reporting of positive drug sensitivity results pose a challenge. Therefore, it is difficult to swiftly and accurately guide the selection of antibiotics. Hence, it is crucial for clinicians to accurately evaluate the condition of children with BSI and rapidly understand pathogen characteristics and drug resistance. This allows for the empirical selection of antibiotics and enhancing the efficacy of infection treatments. Studies on BSI in ICU have been conducted. However, infection control and antimicrobial management policies differ among countries, regions, and hospitals, leading to distinct clinical characteristics among BSI pathogens (Timsit et al. 2020; Xie et al. 2020). The data survey results showed no reports on the characteristics and drug resistance of pathogenic bacteria of BSI in ICU children in the Suzhou area over the last 6 years. Therefore, the purpose of this study was to retrospectively analyze the distribution of pathogenic bacteria, risk factors for death, and drug resistance in children with BSI admitted to the ICU of the Children’s Hospital Affiliated with Suzhou University from January 2016 to December 2021. The aim was to guide clinicians in selecting appropriate empirical treatment schemes by observing the clinical symptoms of children and combining research data before receiving feedback from the laboratory to reduce the problem of antibiotic abuse and the production of multidrug-resistant (MDR) bacteria.

## Materials and methods

### Study site

This study was conducted at the Children’s Hospital of Soochow University, a medical center in East China and the only tertiary children’s hospital in Jiangsu Province. The Children’s Hospital of Soochow University has 1500 beds and serves > 70,000 inpatients and > 2 million outpatients annually. This study was approved by the Ethics Committee of the Children’s Hospital of Soochow University (No. 2021CS158).

### General information

A total of 281 patients with BSI who were admitted to the ICU of the Children’s Hospital of Soochow University between January 2016 and December 2021 were selected for the study. According to the number of pathogenic bacteria isolated from blood culture samples, children with only one type of pathogenic bacteria in the blood culture samples were included in the single infection group (243 cases), while children with ≥ 2 pathogens cultured simultaneously or ≥ 2 pathogens isolated several times in a row were included in the mixed infection group (38 cases).

### Diagnostic criteria for BSI

According to the national diagnostic standard for hospital infection (Diagnostic criteria for nosocomial infection (Trial), 2003), (Ministry of Health of the People’s Republic of China 2003) and the latest definition and diagnostic standard for hospital infection of CDC/NHSN in the USA (CDC/NHSN, 2017), if a patient had isolated pathogenic bacteria from blood samples during hospitalization and exhibited any of the following symptoms or signs: (1) temperature > 38 ℃ or < 36 ℃, accompanied by chills; (2) invasive portals or migratory lesions of pathogens; (3) obvious symptoms of systemic infection and poisoning, but no clear infection focus; and (4) systolic blood pressure lower than 90 mmHg or more than 40 mmHg lower than the original systolic blood pressure. The exclusion criteria were as follows: (1) incomplete case data, (2) elimination of contaminated strains, and (3) repeated detection of the same strains continuously in the same child.

### Strain identification and drug sensitivity test

The blood culture bottles were placed on the instrument for incubation. The positive samples were transferred to the culture plate and incubated at 37 ℃ for 18–24 h (5% CO_2_). The colonies were identified using a mass spectrometer. The drug sensitivity test used both the automatic bacterial detection and analysis system and the Kirby–Bauer (KB) method. The results were evaluated according to the latest standards of the Clinical Laboratory Standardization Association. Extended-spectrum β-lactamases (ESBLs) were detected using an automatic bacterial detection and analysis system. The judgment results were derived from its expert system. The quality control strains, *Escherichia coli* (ATCC 25922), *Pseudomonas aeruginosa* (ATCC 27853), *Staphylococcus aureus* (ATCC 25923 and ATCC 29213), *Enterococcus faecalis* (ATCC 29212), and *Streptococcus pneumoniae* (ATCC 49619), were purchased from the Clinical Testing Center of the National Health Commission.

### Data analysis

SPSS version 20.0 and WHONET 5.6 (WHO Collaborating Centre for Surveillance of Antimicrobial Resistance, Boston, MA, USA) were used to analyze the data. The data are expressed as means and standard deviations ($\overline{\mathrm x}$ ± SD). Frequency data are expressed as number of cases (*n*) and rates (%). The *t*-test and *χ*^2^ test were performed for univariate analysis. Binary logistic regression analysis was used in the multivariable analysis. Statistical significance was set at *P* < 0.05.

## Results

### Basic clinical information

Of the included patients (*N* = 281), 167 were males (59.4%; aged 3.8 ± 4.4 years) and 114 were females (40.6%; aged 3.9 ± 4.1 years). The male-to-female ratio was 1.5:1. The most common diseases were hematological malignancies (*n* = 74, 26.3%), respiratory system diseases (*n* = 57, 20.3%), heart diseases (*n* = 51, 18.2%), and central nervous system diseases (*n* = 39, 13.9%). Pneumonia and central nervous system infections are the most common hospital-acquired infections, while other infections such as urinary tract infections and phlebitis are less common. Of the included patients, 243 were in the single-infection group and 38 were in the mixed-infection group. Children in both groups had similar ages (3.7 ± 4.2 vs 4.6 ± 5.0, *t* = 1.095, *P* = 0.279). There were 259 patients in the survival group and 22 in the death group.

### Annual distribution of pathogenic bacteria [n (%)]

**Fig. 1:**
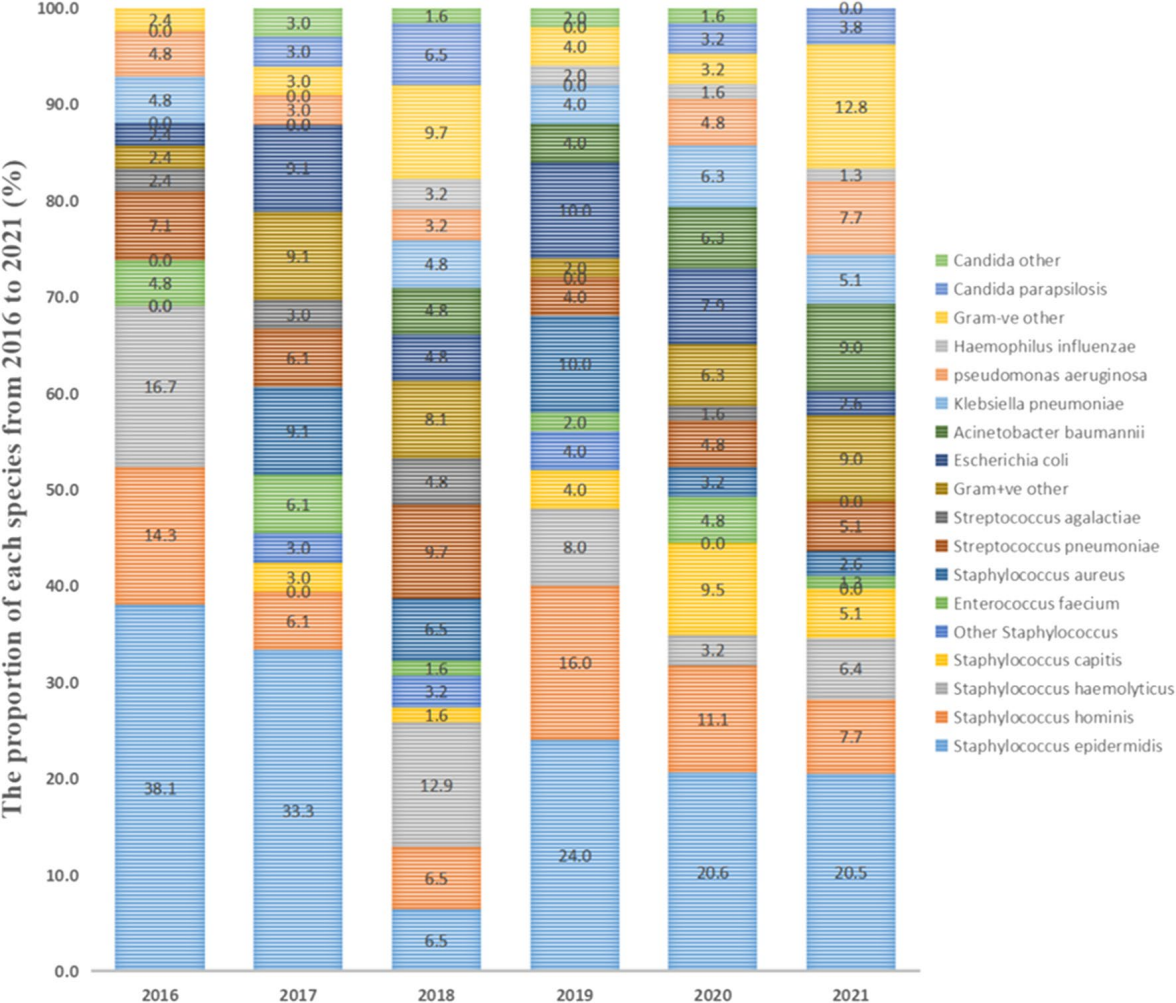
The proportion of the total number of cases each year ascribed to each species

**Fig. 2:**
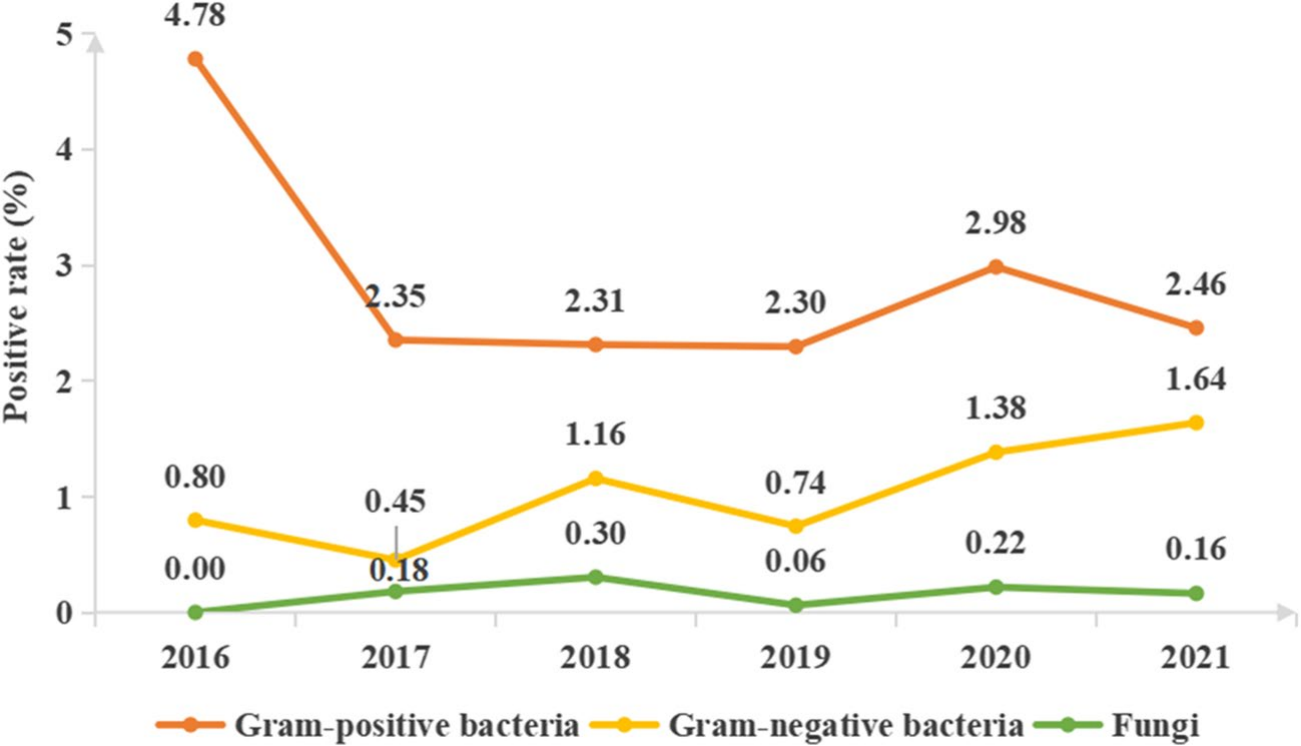
Analysis of the positive rates of BSI in the ICU from 2016 to 2021

As depicted in Fig. 1, Gram-positive bacteria constitute the primary pathogens causing BSI in children admitted to the ICU, consistently exhibiting a higher positive rate compared to Gram-negative bacteria and fungi. Coagulase-negative *Staphylococcus* (CoNS) is the most common BSI in children (see *Supplementary materials Table *S1* for details*). The positive rates of Gram-positive or Gram-negative pathogens and fungi each year are shown in Fig. 2.

### Comparative analysis of clinical characteristics in ICU with BSI [n (%)]

As shown in Table 1, the proportion of male patients aged between 0 and < 3 years with common diseases, such as hematological malignancy, respiratory system disease, heart disease, and central nervous system disease, was higher. Further analysis of mortality risk factors showed a statistically significant difference (*P* < 0.05) in age, length of hospitalization, primary disease, and pathogen type.

**Table 1:** Single factor analysis of children in ICU with BSI [n (%)]

Group		Single infection group (*n* = 243)	Mixed infection group (*n* = 38)	*χ*^2^	*P*	Survival group (*n* = 259)	Death group (*n* = 22)	*χ*^2^	*P*
Gender	Male	144 (59.3)	23 (60.5)	0.022	0.882	155 (59.9)	12 (54.6)	0.236	0.627
	Female	99 (40.7)	15 (39.5)			104 (40.2)	10 (54.5)		
Age	0 ~ < 3Y*	139 (57.2)	20 (52.6)	0.279	0.597	151 (58.3)	8 (36.4)	3.972	0.046
	3 ~ < 14Y	96 (39.5)	16 (42.1)	0.093	0.761	98 (37.8)	14 (63.6)	5.563	0.018
	14 ~ < 18Y	8 (3.3)	2 (5.3)	0.372	0.542	10 (3.9)	0 (0)	0.881	0.348
Length of hospitalization	0 ~ < 15d*	95 (39.1)	3 (7.9)	14.084	< 0.001	85 (32.8)	13 (59.1)	6.162	0.013
	15 ~ < 30d	77 (31.7)	6 (15.8)	3.99	0.046	82 (31.7)	1 (4.6)	7.163	0.007
	30 ~ < 60d	61 (25.1)	21 (55.3)	14.464	< 0.001	78 (30.1)	4 (18.2)	1.397	0.237
	≧60d	10 (4.1)	8 (21.1)	15.724	< 0.001	14 (5.4)	4 (18.2)	5.521	0.019
Main basic diseases	Hematological malignancy	63 (25.9)	11 (29)	0.155	0.694	64 (24.7)	10 (45.5)	4.498	0.034
	Respiratory diseases	45 (18.5)	12 (31.6)	3.466	0.063	55 (21.2)	2 (9.1)	1.85	0.174
	Heart disease	44 (18.1)	7 (18.4)	0.002	0.963	45 (17.4)	6 (27.3)	1.337	0.248
	Central nervous system diseases	37 (15.2)	2 (5.3)	2.729	0.099	39 (15.1)	0 (0)	3.847	0.05
	Traumatic disease	12 (4.9)	4 (10.5)	1.911	0.167	14 (5.4)	2 (9.1)	0.513	0.474
	Digestive system diseases	13 (5.4)	2 (5.3)	0	0.982	15 (5.8)	0 (0)	1.346	0.246
Pathogen type	Gram-positive bacteria	**-**				178 (69)	7 (31.8)	12.281	< 0.001
	Gram-negative bacteria					58 (22.4)	12 (54.6)	11.206	0.001
	Fungi					10 (3.9)	0 (0)	0.881	0.348
	Mixed infection					13 (5.0)	3 (13.6)	2.804	0.094
Survival status	Death	19 (7.8)	3 (7.9)	0	0.987	-			
	Survival	224 (92.2)	35 (92.1)						

*Y means age, d means day

### Multivariable analysis of mortality risk factors among children with BSI

To further explore the meaningful indicators of univariate analysis in Table 1, a multi-factor analysis was performed on the mortality risk factors in children with BSI in the ICU (Table 2).

**Table 2:** Multivariable analysis of meaningful indicators

Variable	*B*	*SE*	*Waldχ*^2^	*P*	OR (95% CI)
Length of hospitalization	− 0.136	0.260	0.272	0.602	0.873 (0.524–1.145)
Main basic diseases	− 2.388	0.922	6.709	0.01	0.092 (0.602–1.125)
Age	0.455	0.388	1.374	0.241	1.575 (0.737–3.369)
Mixed infection	0.30	0.699	0.002	0.965	1.031 (0.262–4.054)

*OR, the odds ratio; 95% CI, the 95% confidence interval

### Analysis of resistance of major Gram-positive bacteria to commonly used antibiotics

The resistance rates of *Staphylococcus* to penicillin and erythromycin were high. None of the detected *Staphylococcus* strains were resistant to quinuputin/dafopratin, linezolid, vancomycin, teicoplanin, or tigecycline. Table 3 shows the drug resistance analysis of the main Gram-positive bacteria to common antibiotics.

**Table 3:** Analysis of resistance of the main gram-positive bacteria to common antibiotics [n (%)]

Types of antibiotics		*Staphylococcus epidermidis* (*n* = 72)	*Staphylococcus haemolyticus* (*n* = 26)	*Staphylococcus aureus* (*n* = 16)	Streptococcus pneumoniae (*n* = 20)	*Enterococcus faecium* (*n* = 10)
Macrolides	Erythromycin	60 (83.3)	24 (92.3)	10 (62.5)	20 (100)	-
	Clindamycin	27 (37.5)	15 (57.7)	7 (43.8)	19 (95)	-
β-lactamides	Penicillin	70 (97.2)	25 (96.2)	16 (100)	0	9 (90)
	Oxacillin	60 (83.3)	24 (92.3)	4 (25)	-	-
	Amoxicillin	-	-	-	2 (10)	-
	Ampicillin	-	-	-	-	10 (100)
	Cefoxitin	61 (84.7)	24 (92.3)	4 (25)	-	-
	Cefotaxime	-	-	-	3 (15)	-
Streptoyangmycin	Quinuptin/Dafopudin	0 (0)	0 (0)	0 (0)	13 (50)	0 (0)
Rifamycins	Rifampicin	7 (9.72)	4 (15.4)	0 (0)	0 (0)	-
Sulfonamides	Compound sulfamethoxazole	40 (55.6)	8 (30.8)	0 (0)	15 (75)	-
Quinolones	Ciprofloxacin	13 (18.1)	16 (61.5)	0 (0)	-	4 (40)
	Levofloxacin	17 (23.6)	13 (50)	1 (6.3)	0 (0)	3 (30)
	Moxifloxacin	2 (2.8)	8 (30.8)	1 (6.3)	-	-
Aminoglycosides	Gentamicin	10 (13.9)	12 (46.2)	0 (0)	-	-
Chloramphenicols	Chloramphenicol	-	-	-	2 (10)	-
Tetracyclines	Tetracycline	15 (20.8)	9 (34.6)	3 (18.8)	16 (80)	3 (30)
	Minocycline	-	-	-	-	5 (50)

“-”, this means it is not detected

### Analysis of resistance of the main Gram-negative bacteria to common antibiotics [n (%)]

Compared with *Escherichia coli*, *Klebsiella pneumoniae* showed lower rates of resistance to aztreonam, cefuroxime, cefotaxime, ceftriaxone, ciprofloxacin, levofloxacin, gentamycin, and compound sulfamethoxazole. The resistance rates of *Acinetobacter baumannii* and *Pseudomonas aeruginosa* to antibiotics are shown in Table 4.

**Table 4:** Analysis of resistance of the main Gram-negative bacteria to common antibiotics [n (%)]

Types of antibiotics		*Escherichia coli* (*n* = 19)	*Klebsiella pneumoniae* (*n* = 15)	*Acinetobacter baumannii* (*n* = 16)	*Pseudomonas aeruginosa* (*n* = 14)
ESBLs producing strains		11 (57.9)	8 (53.3)	-	-
Carbapenems	Imipenem	1 (5.3)	3 (20)	13 (81.3)	6 (42.7)
Monobactams	Aztreonam	7 (36.8)	2 (13.3)	-	3 (21.4)
Penicillins	Ampicillin	15 (79)	14 (93.3)	-	-
Enzyme inhibitor complex	Ampicillin/Sulbactam	12 (63.2)	13 (86.7)	12 (75)	-
	Cefoperazone/Sulbactam	3 (15.8)	4 (26.7)	12 (75)	3 (21.4)
	Piperacillin/Tazobactam	1 (5.3)	4 (26.7)	12 (75)	1 (7.1)
Extended spectrum cephalosporin	Cefazolin	10 (52.6)	8 (53.3)	-	-
	Cefuroxime	11 (57.9)	7 (46.7)	-	-
	Cefotaxime	8 (42.1)	6 (40)	-	-
	Cefazoxime	7 (36.8)	6 (40)	-	-
	Cefotetan	1 (5.3)	4 (26.7)	-	-
	Ceftriaxone	11 (57.9)	8 (53.3)	12 (75)	-
	Ceftazidime	5 (26.3)	5 (33.3)	12 (75)	1 (7.1)
	Cefepime	5 (26.3)	5 (33.3)	12 (75)	2 (14.3)
Cephamicins	Cefoxitin	2 (10.5)	3 (20)	-	-
Quinolones	Ciprofloxacin	8 (42.1)	5 (33.3)	12 (75)	0 (0)
	Levofloxacin	8 (42.1)	2 (13.3)	7 (43.8)	0 (0)
Aminoglycosides	Amikacin	0 (0)	1 (6.7)	12 (75)	0 (0)
	Gentamicin	11 (57.9)	4 (26.7)	12 (75)	0 (0)
	Tobramycin	2 (10.5)	2 (13.3)	11 (68.8)	0 (0)
Sulfonamides	Compound sulfamethoxazole	11 (57.9)	5 (33.3)	12 (75)	-
Tetracyclines	Minocycline	-	-	6 (37.5)	-

“-”, it indicates that the strain is not tested or is naturally resistant to the antibiotic

### Detection of MDR bacteria

As shown in Table 5, 27 common MDR bacteria were isolated, including 23 strains of carbapenem-resistant Gram-negative bacteria and four strains of methicillin-resistant *Staphylococcus aureus* (MRSA). Vancomycin-resistant Enterococcus (VRE) strains were not detected.

**Table 5:** Detection of MDR bacteria

MDR bacteria	2016	2017	2018	2019	2020	2021	Total
CRAB	0	0	2	1	4	6	13
CRPA	1	0	0	0	1	4	6
CRKP	0	0	1	1	0	1	3
CREO	0	0	0	0	1	0	1
MRSA	0	0	2	1	0	1	4
VRE	0	0	0	0	0	0	0
Total	1	0	5	3	6	12	27

## Discussion

BSI can manifest as bacteremia or progress to sepsis, a common occurrence among critically ill children in the ICU. The incidence of BSI has increased in recent years. Children might exhibit only transient infection symptoms, while others may develop severe sepsis and shock, often leading to a poor prognosis. Blood culture is currently the gold standard for the diagnosis of BSI, and antibiotics can be selected based on the results of bacterial culture and drug sensitivity in clinical practice (Soedarmono et al. 2022). It has been reported that in-hospital mortality caused by severe BSI is as high as 30–60%, which exceeds the total mortality caused by breast cancer, acquired immunodeficiency syndrome, and prostate cancer (Martínez Pérez-Crespo et al. 2021). Every hour that treatment is delayed increases the mortality of children by 7.6% (Kumar et al. 2006). International guidelines suggest that effective antibiotics should be injected intravenously within 1 h of sepsis diagnosis (Dellinger et al. 2013). Therefore, it is necessary to summarize and analyze the pathogen distribution, related risk factors, and drug sensitivity results of BSI in ICU children to help clinicians select appropriate empirical treatment plans, improve the prognosis of children with sepsis, and reduce BSI mortality.

In our study, 328 pathogenic strains were isolated, including Gram-positive bacteria (68%, 223/328), Gram-negative bacteria (27.7%, 91/328), and fungi (4.3%, 14/328). The main Gram-positive bacteria were CoNS (47.86%, 157/328), *Streptococcus pneumoniae* (6.10%, 20/328), and *Staphylococcus aureus* (4.9%, 16/328). The main Gram-negative bacteria were *Escherichia coli* (5.8%, 19/328), *Acinetobacter baumannii* (4.9%, 16/328), *Klebsiella pneumoniae* (4.6%, 15/328), and *Pseudomonas aeruginosa* (4.3%, 14/328). The fungi type identified was mainly *Candida parapsilosis* (3.1%, 10/328). As shown in Fig. 1, the positive blood culture rates from 2016 to 2021 are 5.6%, 3%, 3.8%, 3.1%, 4.6%, and 4.3%, respectively. Gram-positive bacteria, represented by CoNS, are the main pathogens that cause BSI in the ICU, and their rate has always been higher than that of Gram-negative bacteria and fungi. A study comparing the pathogens of BSI between children and adults in the ICU found that most adults had Gram-negative bacteria, while the children had CoNS (Zhang et al. 2021). An increasing number of studies have shown that cases of BSI caused by Gram-positive bacteria are on the rise (Santella et al. 2020; Wang et al. 2021; Dambroso-Altafini et al. 2022). In contrast, some studies have shown that Gram-negative bacteria are the main pathogens (Amanati et al. 2021; Zain et al. 2022). Variations in BSI pathogen detection could be attributed to factors, such as timing, geographical location, and study objective. As such, the results solely reflect the situation within the research institution during a specific period. Additionally, similar to previous studies, the most common Gram-negative bacterium causing BSI in this study was *Escherichia coli* (Zain et al. 2022; Hu et al. 2022). In recent years, the incidence of BSI caused by fungi has increased, with Candida being the most common fungi (Lee et al. 2021). In our study, however, the fungal infection rate in children was lower compared with bacterial infections, and *Candida parapsilosis* emerged as the main infectious pathogen.

Among the 281 patients with BSI, 243 were infected by one pathogen (86.5%, 243/281) and 38 were infected by mixed pathogens (13.5%, 38/281), which is close to the mixed infection rate reported in the previous studies (6–13%) (Kiani et al. 1979; Rello et al. 1993; Lin et al. 2010). In the mixed-infection group, dual infections with two pathogens constituted 11.03%, triple-pathogen infections accounted for 1.8%, and quadruple-pathogen infections accounted for 0.7%. When comparing the clinical data of children in the single and mixed-infection groups, we found that there was a significant difference in the length of hospitalization between the two groups (*P* < 0.05). Mixed infections are the most complex and serious type of infection associated with sepsis. The treatment efficacy is suboptimal and the prognosis is often poor (Lin et al. 2010). Early diagnoses, along with timely and effective antibiotic therapy, are keys to improving the prognosis of pediatric patients with sepsis.

To further explore the risk factors for death from BSI, 281 children with BSI in the ICU were divided into a survival group (*n* = 259) and a death group (*n* = 22). The length of hospitalization, age, primary diseases, and pathogen type were significantly different between the two groups (*P* < 0.05). Binary logistic regression analysis revealed that the primary disease, notably hematological malignancies, was an independent risk factor for mortalities (Table 2). Of the 281 children diagnosed with BSI in the ICU, 22 died, resulting in a mortality rate of 7.8%. The mortality rate among the children in the mixed-infection group was 7.89% (3/38), which was slightly higher than that in the single-infection group (7.82% (19/243)). Initially, most mixed infections are considered single-pathogen infections, leading to inadequate empirical drug treatment. This inadequacy exacerbates the patient’s conditions, prolongs hospital stay, and increases mortality rates (Shen et al. 2015). Therefore, it is critical to identify children with a high risk of mixed infections, ascertain the suspected source of infection, and promptly identify the predominant pathogens. This will help understand the antimicrobial resistance patterns within local medical institutions.

In order to remind doctors to understand the current situation of pathogen resistance to certain antibiotics in the local area and to use antibiotics reasonably, we have conducted statistics on the resistance of major pathogens in recent years. The most common Gram-positive bacteria include *Staphylococcus epidermidis*, *Staphylococcus haemolyticus*, *Streptococcus pneumoniae*, *Staphylococcus aureus*, and *Enterococcus faecium*. As shown in Table 3, the resistance rates of Staphylococcus to penicillin and erythromycin were high. Vancomycin-resistant strains have also been reported (Fournier et al. 2013). None of the Gram-positive bacteria were resistant to linezolid, vancomycin, teicoplanin, or tigecycline in our study. CoNS belong to the normal flora of the human skin and mucosal tissue. Numerous reports highlight CoNS’s association with infectious diseases, particularly catheter-related BSI (May et al. 2014; Matarrese et al. 2021). Most CoNS strains are methicillin-resistant coagulase-negative *Staphylococcus* (MRCoNS). In total, 61 strains of methicillin-resistant coagulase-negative *Staphylococcus epidermidis* (84.7%, 61/72) and 24 strains of methicillin-resistant coagulase-negative *Staphylococcus haemolyticus* (92.3%, 24/26) were isolated in this study, both of which showed multiple drug resistance. This is consistent with results from a previous study (Peng et al. 2021). Compared with *Staphylococcus epidermidis* and *Staphylococcus haemolyticus*, *Staphylococcus aureus* showed a lower drug resistance rate to some β-lactamides and quinolone antibiotics, and they were 100% sensitive to compound sulfamethoxazole, gentamicin, and tigecycline. Four strains of MRSA and five strains of *Staphylococcus aureus* with positive D results were detected. MRSA is resistant to all β-lactamide antibiotics. *Streptococcus pneumoniae* was more than 95% resistant to macrolide antibiotics (erythromycin and clindamycin) and more than 50% resistant to tetracycline, compound sulfamethoxazole, and quinuputin/dafopratin. However, the drug resistance rate to amoxicillin was low (only 10%). *Enterococcus faecium* was 100% sensitive to quinuputin/dafopratin, linezolid, vancomycin, teicoplanin, and tigecycline, consistent with a recent study (Tian 2022). The resistance rate of *Enterococcus faecium* to penicillin was as high as 90%, whereas the resistance rate to some quinolone antibiotics was low (30–40%). In our study, the resistance rate of the isolated Gram-positive bacteria to quinolones (ciprofloxacin, levofloxacin, and moxifloxacin) and aminoglycoside antibiotics (gentamicin) was lower than that of other antibiotics, possibly due to the influence of quinolones on bone development, the nephrotoxicity, and ototoxicity of aminoglycoside antibiotics and their reduced use in children.

Of the 22 children who died during the study period, 14 were infected with Gram-negative bacteria (63.6%, 14/22). This highlights the critical need to focus on children infected with Gram-negative bacteria in ICUs. Studies have shown that BSI caused by Gram-negative bacteria is an independent risk factor for high mortality in ICU (Dat et al. 2018). Ninety-one strains of Gram-negative bacteria were isolated, including *Escherichia coli*, *Acinetobacter baumannii*, *Klebsiella pneumoniae*, and *Pseudomonas aeruginosa*. The drug sensitivity results for the four bacterial strains are presented in Table 4. The composition ratios of the ESBLs-producing strains of *Escherichia coli* and *Klebsiella pneumoniae* were 57.9% (11/19) and 53.3% (8/15), respectively. However, the drug resistance rate of *Klebsiella pneumoniae* to imipenem was higher than that of *Escherichia coli* (20% vs.5.6%). Studies have shown that the incidence of BSI caused by carbapenem-resistant *Klebsiella pneumoniae* (CRKP) is increasing (Stein et al. 2019; Guo et al. 2023). According to data from the *National Drug Resistance Monitoring Network (*http://www.carss.cn/*)*, the isolation rate of CRKP among children in China increased from 3.0 to 20.9% from 2005 to 2017, which was significantly higher than that among adults (Wang et al. 2020). Compared with *Escherichia coli*, *Klebsiella pneumoniae* has a lower rate of resistance to aztreonam, cefuroxime, cefotaxime, ceftriaxone, ciprofloxacin, levofloxacin, gentamycin, and cotrimoxazole. The resistance rate of *Acinetobacter baumannii* to various antibiotics was higher than 75%, whereas the resistance rates to levofloxacin and minocycline were lower at 43.8% and 37.5%, respectively. These results highlight the concerning drug resistance of *Acinetobacter baumannii*, revealing a limited array of effective drug options. A combination of tegacycline-based drugs for the treatment of severe infections caused by *Acinetobacter baumannii* is the more commonly used regimen. Recently, our research group conducted a study on the carbapenem resistance and virulence of *Acinetobacter baumannii* and analyzed the cause of its multiple drug resistance (Zhu et al. 2022). *Pseudomonas aeruginosa* is sensitive to commonly used clinical anti-pseudomonas drugs, with a resistance rate of 21.4% to aztreonam, 21.4% to cefoperazone/sulbactam, 7.1% to piperacillin/tazobactam, 7.1% to ceftazidime, and 21.4% to cefepime. Pseudomonas aeruginosa was 100% resistant to quinolones (ciprofloxacin and levofloxacin) and aminoglycosides (gentamicin, tobramycin, and amikacin).

Among the 328 pathogenic bacteria identified in this study, 27 common MDR bacteria were isolated, including 13 strains of carbapenem-resistant *Acinetobacter baumannii* (CRAB), six strains of carbapenem-resistant *Pseudomonas aeruginosa* (CRPA), three strains of carbapenem-resistant *Klebsiella pneumoniae* (CRKP), one strain of carbapenem-resistant *Escherichia coli* (CREO), and four strains of MRSA. VRE strains were not detected. More MDR bacteria strains were detected in 2021. In this study, the proportion of CRAB is high (48.2%; 13/27), which is consistent with another study (Bedenić et al. 2023). The proportion of patients with CRPA was the second highest, accounting for 22.2% (6/27). Among the 22 children who died, four were infected with CRAB and two with CRPA. Carbapenem-resistant *Enterobacter* (CRE) can be found in the urine, respiratory tract, feces, blood, and other samples (Kotb et al. 2020; Sexton et al. 2022; Xiong et al. 2023). Four CRE strains were identified in this study. These results underscore the need to focus on BSI caused by CR bacteria. Studies have demonstrated that the main cause of the resistance of pathogenic bacteria to carbapenem antibiotics in children is the production of metalloenzymes (class B) (Buys et al. 2016). Currently, the most effective antibiotic combination against CRE is polymyxin coupled with tigecycline (Vanegas et al. 2016). However, tigecycline is rarely used in children because it causes tooth staining. Polymyxins alone are a relatively safe treatment for children. Further analysis of the clinical treatment effects, such as the length of hospitalization and whether there is improvement in these MDR-infected patients, is necessary. Among the 22 children who died, 17 had hematological malignancies (> 50% of the proportion). Therefore, it is essential to focus on BSI in children with hematological malignancies.

In summary, the pathogens causing BSI in children admitted to the ICU in the past 6 years are mainly Gram-positive bacteria such as CoNS. *Escherichia coli* is the most common Gram-negative bacterium. As such, continuous monitoring of blood cultures in critically ill children with BSI is necessary. This entails focusing on detecting MDR bacteria, improving antibiotic application and management, and enhancing hospital infection prevention and control measures. This study has some limitations. Due to the small variation in the number of pathogens detected each year, an analysis of the annual changes in antimicrobial resistance could not be performed. We plan to expand the study period in the next study to more accurately analyze the changes in drug resistance rate.

## Supplementary Information

Supplementary File 1 (DOCX 25.1 kb)

## Data Availability

The datasets used and/or analyzed during the current study are available from the corresponding author on reasonable request.

## Declarations

### Disclaimer

The views, opinions, assumptions, or any other information set out in this article are solely those of the authors and should not be attributed to the funders or any other person connected with the funders.

## Abbreviations

*KB*: Kirby–Baue
*ICU*: Intensive care unit
*MDR*: Multidrug resistant
*BSI*: Bloodstream infection
*VRE*: Vancomycin-resistant *Enterococcus*
*CoNS*: Coagulase-negative staphylococci
*ESBLs*: Extended-spectrum β-lactamases
*CRE*: Carbapenem-resistant enterobacter
*CREO*: Carbapenem-resistant *Escherichia coli*
*CRKP*: Carbapenem-resistant *Klebsiella pneumoniae*
*CRAB*: Carbapenem-resistant *Acinetobacter baumannii*
*CRPA*: Carbapenem-resistant *Pseudomonas aeruginosa*
*MRSA*: Methicillin-resistant *Staphylococcus aureus*
*MRCoNS*: Methicillin-resistant coagulase-negative *staphylococcus*
