# Supplementary material for: Epidemiology and drug resistance analysis of bloodstream infections in an intensive care unit from a children’s medical center in Eastern China for six consecutive years
Source: Int Microbiol. 2024 Jan 18;27(5):1345–55. doi: 10.1007/s10123-024-00481-2 (PMC11452477; doi:10.1007/s10123-024-00481-2)
Supplement: Supplementary file 1 — (DOCX 25.1 kb) [file 10123_2024_481_MOESM1_ESM.docx]

Supplementary materials

**Table S1 Pathogen distribution of BSI from 2016 to 2021**

| Type | 2016 | 2017 | 2018 | 2019 | 2020 | 2021 | Total | Percentage (%) |
| --- | --- | --- | --- | --- | --- | --- | --- | --- |
| **Gram-positive bacteria** | 36 | 26 | 38 | 37 | 41 | 45 | 223 | 68 |
| *Staphylococcus epidermidis* | 16 | 11 | 4 | 12 | 13 | 16 | 72 | 22 |
| *Staphylococcus hominis* | 6 | 2 | 4 | 8 | 7 | 6 | 33 | 10.1 |
| *Staphylococcus haemolyticus* | 7 | 0 | 8 | 4 | 2 | 5 | 26 | 7.9 |
| *Staphylococcus capitis* | 0 | 1 | 1 | 2 | 6 | 4 | 14 | 4.3 |
| *Staphylococcus warneri* | 0 | 1 | 0 | 1 | 1 | 1 | 4 | 1.2 |
| *Staphylococcus caprae* | 0 | 1 | 1 | 0 | 1 | 0 | 3 | 0.9 |
| *Staphylococcus aureus* | 0 | 3 | 4 | 5 | 2 | 2 | 16 | 4.9 |
| Other *Staphylococcus* | 0 | 1 | 2 | 2 | 0 | 0 | 5 | 1.5 |
| *Streptococcus pneumoniae* | 3 | 2 | 6 | 2 | 3 | 4 | 20 | 6.1 |
| *Streptococcus agalactiae* | 1 | 1 | 3 | 0 | 1 | 0 | 6 | 1.8 |
| *Streptococcus mitis* | 1 | 1 | 0 | 0 | 2 | 0 | 4 | 1.2 |
| *Streptococcus oralis* | 0 | 0 | 1 | 0 | 0 | 1 | 2 | 0.6 |
| *Streptococcus pyogenes* | 0 | 0 | 1 | 0 | 0 | 0 | 1 | 3.9 |
| Other *Streptococcus* | 0 | 0 | 1 | 0 | 0 | 3 | 4 | 1.2 |
| *Enterococcus faecium* | 2 | 2 | 1 | 1 | 3 | 1 | 10 | 3.1 |
| *Enterococcus faecalis* | 0 | 0 | 1 | 0 | 0 | 2 | 3 | 0.9 |
| **Gram-negative bacteria** | 6 | 5 | 19 | 12 | 19 | 30 | 91 | 27.7 |
| *Escherichia coli* | 1 | 3 | 3 | 5 | 5 | 2 | 19 | 5.8 |
| *Acinetobacter baumannii* | 0 | 0 | 3 | 2 | 4 | 7 | 16 | 4.9 |
| *Klebsiella pneumoniae* | 2 | 0 | 3 | 2 | 4 | 4 | 15 | 4.6 |
| *Pseudomonas aeruginosa* | 2 | 1 | 2 | 0 | 3 | 6 | 14 | 4.3 |
| *Haemophilus influenzae* | 0 | 0 | 2 | 1 | 1 | 1 | 5 | 1.5 |
| *Burkholderia cepacia* | 0 | 0 | 2 | 0 | 0 | 1 | 3 | 0.9 |
| *Stenotrophomonas maltophilia* | 0 | 0 | 1 | 0 | 0 | 2 | 3 | 0.9 |
| *Acinetobacter nosocomialis* | 0 | 0 | 1 | 0 | 0 | 1 | 2 | 0.6 |
| *Acinetobacter pittei* | 0 | 0 | 0 | 1 | 0 | 1 | 2 | 0.6 |
| *Morganella morganii* | 0 | 1 | 0 | 0 | 0 | 0 | 1 | 0.3 |
| *Klebsiella aerogenes* | 0 | 0 | 0 | 0 | 0 | 1 | 1 | 0.3 |
| *Citrobacter klebsiella* | 0 | 0 | 0 | 0 | 1 | 0 | 1 | 0.3 |
| *Acinetobacter junii* | 0 | 0 | 1 | 0 | 0 | 0 | 1 | 0.3 |
| *Serratia marcescens* | 0 | 0 | 0 | 0 | 0 | 1 | 1 | 0.3 |
| *Pantoea agglomerans* | 0 | 0 | 0 | 1 | 0 | 0 | 1 | 0.3 |
| *Sphingomonas pseudohaemolyticus* | 1 | 0 | 0 | 0 | 0 | 0 | 1 | 0.3 |
| *Acinetobacter lwoffii* | 0 | 0 | 0 | 0 | 0 | 1 | 1 | 0.3 |
| *Salmonella* | 0 | 0 | 0 | 0 | 0 | 1 | 1 | 0.3 |
| *Enterobacter cloacae* | 0 | 0 | 1 | 0 | 0 | 0 | 1 | 0.3 |
| *Proteus mirabilis* | 0 | 0 | 0 | 0 | 1 | 0 | 1 | 0.3 |
| *Klebsiella oxytoca* | 0 | 0 | 0 | 0 | 0 | 1 | 1 | 0.3 |
| **Fungi** | 0 | 2 | 5 | 1 | 3 | 3 | 14 | 4.3 |
| *Candida parapsilosis* | 0 | 1 | 4 | 0 | 2 | 3 | 10 | 3.1 |
| *Candida tropicalis* | 0 | 0 | 1 | 0 | 1 | 0 | 2 | 0.6 |
| *Candida albicans* | 0 | 1 | 0 | 1 | 0 | 0 | 2 | 0.6 |
| **Total** | 42 | 33 | 62 | 50 | 63 | 78 | 328 | 100 |
| **The number of specimens submitted** | 753 | 1105 | 1643 | 1612 | 1374 | 1831 | 8318 | - |
